# Geographic variability of fatal road traffic injuries in Spain during the period 2002–2004: an ecological study

**DOI:** 10.1186/1471-2458-7-266

**Published:** 2007-09-26

**Authors:** Francisco Rivas-Ruiz, Emilio Perea-Milla, Alberto Jimenez-Puente

**Affiliations:** 1 Unidad de Apoyo a la Investigación (Red IRYSS), Hospital Costa del Sol, Ctra Nacional 340, km 187, 29600 Marbella, Spain; 2 Unidad de Evaluación (Red IRYSS), Hospital Costa del Sol, Ctra Nacional 340, km 187, 29600 Marbella, Spain

## Abstract

**Background:**

The aim of the present study is to describe the inter-province variability of Road Traffic Injury (RTI) mortality on Spanish roads, adjusted for vehicle-kilometres travelled, and to assess the possible role played by the following explicative variables: sociodemographic, structural, climatic and risk conducts.

**Methods:**

An ecological study design was employed. The mean annual rate of RTI deaths was calculated for the period 2002–2004, adjusted for vehicle-kilometres travelled, in the 50 provinces of Spain. The RTI death rate was related with the independent variables described above, using simple and multiple linear regression analysis with backward step-wise elimination. The level of statistical significance was taken as p < 0.05.

**Results:**

In the period 2002–2004 there were 12,756 RTI deaths in Spain (an average of 4,242 per year, SD = 356.6). The mean number of deaths due to RTI per 100 million vehicle-kilometres (mvk) travelled was 1.76 (SD = 0.51), with a minimum value of 0.66 (in Santa Cruz de Tenerife) and a maximum of 3.31 (in the province of Lugo). All other variables being equal, a higher proportion of kilometres available on high capacity roads, and a higher cultural and education level were associated with lower death rates due to RTI, while the opposite was true for the rate of alcohol consumers and the road traffic volume of heavy vehicles. The variables included in the model accounted for 55.4% of the variability in RTI mortality.

**Conclusion:**

Adjusting RTI mortality rates for the number of vehicle-kilometres travelled enables us to identify the high variability of this cause of death, and its relation with risk factors other than those inherent to human behaviour, such as the type of roads and the type of vehicles using them.

## Background

Motorization has improved people's lives and that of society in general, but at a high price. In 2002 it was estimated that road traffic injuries (RTI) worldwide caused 1.2 million deaths and 50 million injuries, at a monetary cost of 518 billion dollars [1], equivalent to 2% of the GDP of all the world's developed countries [2]. In 2002, RTI were the ninth greatest cause of years of potential life lost (YPLL), and this problem will only get worse unless effective action is taken – thus, they are expected to become the third most important factor in YPLL by 2020 [3].

In developed countries, highways are mainly used by car drivers, and the latter represent most of the deaths caused in road traffic. The 15–44 years age group accounts for half of all deaths [4,5], and for 70% of YPLL [6]. By sexes, the RTI death rate is higher among men, as they are exposed to the danger for longer periods than women due to differences in their occupations and access to vehicle use [7]. In general, young males are involved in more collisions and have a higher road fatality rate because of their risk taking behaviour when driving. [8].

Worldwide, the following factors are most clearly associated with RTI: the type of vehicle and its maintenance condition; the design of highway infrastructure; meteorological conditions; risk conduct by drivers, such as alcohol intake, drug consumption, excessive speed, lack of sleep, driver distraction and neglect of security measures [9,10]. In Spain, in studies with individual data, the risk factors associated with RTI are excessive and inappropriate speed, driving under the influence of alcohol and somnolence [11,12].

Actions aimed at reducing the RTI rate seek to modify the risk practices of drivers and passengers. Thus, it is reported that the control of vehicle speeds by means of security cameras is associated with a reduction of 60% in the number of deaths occurring on roads where collision rates are particularly high [13]. It is considered self-evident that road mortality is reduced by the implementation of controls on alcohol-affected driving [14], by the compulsory use of helmets by motorcyclists [15], and by the use of seat belts in cars [16]. Thus, it is estimated that if all the road safety laws currently applicable in the European Union were complied with, deaths and serious injuries would be reduced by 50% [13]. Therefore, about half of RTI mortality is associated with other factors. Despite the obvious improvement achieved by policies to reduce RTI mortality in developed countries [17], road safety strategies remain entrenched within a blame-attaching model, with the causes of RTI basically being attributed to human conduct [18]. The road safety policies arising from such a standpoint, hence, are based on education, supervision and law enforcement.

However, the probability of suffering a RTI is not exclusively inherent to the human factor; we must also consider the considerable influence of the type of roads used and the type of traffic that is driven on these roads.

The bibliography consulted contains no indication concerning the most appropriate denominator to be used in analyses of the incidence of collisions, comparable to the epidemiologic concept of person time at risk. The indicators utilised elsewhere in analysing the variability in RTI mortality include measures adjusted for the population or the total number of vehicles [19,20]. A valid approach, would be to take into consideration the vehicle-kilometres travelled. The adjusted value per vehicle-kilometres travelled has been used in a descriptive way in several studies [21,22]. It is a very suitable indicator for a descriptive study, as defined by Farchi et al. [23]. The concept of person-time in risk is commonly used in descriptive and analytical epidemiology, as a denominator enabling the description and comparison of diverse exposures in terms of 'rate of morbi-mortality' or 'rate of change per unit of time'. The advantage of this concept is that is does not require the analysis of closed cohorts, but instead accepts open, dynamic cohorts that items may enter or leave, and that even allow changes in levels of exposure [24]. The concept of vehicle-kilometres travelled is the best possible approximation to that of person-time in risk.

In addition, the special feature of the causative events in road traffic collisions is that there is no linear association between the effect and the exposure to road traffic. RTIs are modulated by behavioural and environmental factors, and this requires a stratified or multivariate analysis of the underlying dimensions. Many studies have performed this type of analysis, but to the best of our knowledge none have done so on the basis of data adjusted per vehicle-kilometres travelled.

Analysis of variability is an important element in the context of an epidemiological analysis of health problems, prior to a study of explicative factors.

The aim of the present study is to describe the inter-province variability of RTI mortality on Spanish roads, adjusted for vehicle-kilometres travelled, and to assess the possible role played by the following explicative variables: sociodemographic, structural, climatic and risk conducts.

## Methods

Ecological study of spatial correlation; the unit of analysis applied was the 50 provinces of Spain.

From the record of road deaths supplied by the Spanish Directorate General of Traffic (DGT), which publishes the RTI deaths occurring during the previous 30 days, the mean annual rate of RTI deaths was calculated for the period 2002–2004, adjusted for vehicle-kilometres travelled, in the 50 provinces of Spain (Source: Spanish Ministry for Infrastructure).

The independent variables applied in the analysis were: a) percentage of high capacity roads (motorways and dual carriageways); percentage of heavy vehicles (over 3500 kg); c) percentage of the population aged over 16 years that consume alcohol; d) mean annual precipitation (mm) during the period 2002–2004; e) index of the cultural and educational level, produced by the Research Dept. of La Caixa, using 4 variables for the educational level (rate of illiteracy or unschooled, rate of secondary school educational attainment, rate of university educational attainment, and rate of secondary school attendance) and 6 variables for the cultural level (audience figures for TV news bulletins, cinemas, internet, rate of library users, total adult education-attendance rate and total special-regime education users); f) index of income, produced by the Research Dept. of la Caixa, based on the income per capita recorded for 2001. For all the indexes analysed, the original values were transformed into index numbers with respect to a mean national value of 100. The division into levels (low, medium and high) corresponds to the distribution by terciles. The source for the information on the types of roads and the percentage of heavy vehicles was the Ministry for Infrastructure (2004), and that for annual precipitation was the Directorate General of the Spanish National Institute of Meteorology, while the data on indexes (income and educational-cultural levels) were obtained from the 2004 Anuario Social (Social Yearbook) published by the Research Dept. of La Caixa [25].

In this descriptive analysis, the weighted mean (adjusted for vehicle-kilometres travelled per province) and the standard deviation (SD) of the study variables were calculated, together with the following statistics of variability for the mean annual death rate from RTI: a) ratio of variation (between the maximum and minimum values), excluding percentiles 5 and 95 (RV_5–95_), and the weighted coefficient of variation (ratio of the standard deviation between provinces to the mean inter-province value, weighted by the size of each area), excluding percentiles 5 and 95 (CVw_5–95_).

The RTI death rate was related with the independent variables described above, using simple and multiple linear regression analysis with backward step-wise elimination. The regression coefficients (beta -β-) together with the corresponding 95% confidence intervals (CI), and the coefficient of determination (R2) are shown. The level of statistical significance was taken as p < 0.05.

The fulfilment of independence, homoscedasticity, and of normality in the multiple linear regression model were tested. Also investigated were the possibility of interaction between the variables, and the absence of colinearity, by calculating the factors of inflation of the variance and by principal component analysis.

## Results

According to the records of the DGT, in the period 2002–2004 there were 12,756 RTI deaths in Spain (an average of 4,242 per year, SD = 356.6). The mean number of deaths due to RTI per 100 million vehicle-kilometres (mvk) travelled was 1.76 (SD = 0.51), with a minimum value of 0.66 (in Santa Cruz de Tenerife) and a maximum of 3.31 (in the province of Lugo). According to the analysis of variability, the RV5–95 was 2.58, and the CVw5–95 was 0.25 (Table 1).

**Table 1:** Mean provincial values for the study variables for the period 2002–2004

**Province**	Deaths × 100 mkr *	**% High capacity roads**	**% Heavy vehicles**	**% Consumers of alcohol**	**Mean precipitacion (mm)**	**Educational and culture level**	**Income**
**Lugo**	3.1	1.8	14.8	50.0	1020.5	Low	Low
**Huesca**	3.0	2.5	13.6	50.5	509.2	Medium	Medium
**Avila**	2.8	3.0	17.1	41.9	421.9	Low	Medium
**Castellón/Castelló**	2.8	9.7	21.1	51.7	598.6	Low	Medium
**Rioja (La)**	2.7	7.9	19.4	43.4	391.1	Medium	High
**Teruel**	2.7	1.2	21.2	37.3	516.2	Low	Medium
**Cáceres**	2.5	4.8	16.1	35.6	676.9	Low	Low
**Ourense**	2.5	4.6	13.7	50.9	923.8	Low	Low
**Salamanca**	2.5	2.6	21.2	38.7	308.1	Medium	Low
**Soria**	2.5	1.6	28.2	41.5	592.9	Medium	Medium
**Almería**	2.4	12.0	13.6	47.8	178.6	Low	Medium
**Lleida**	2.4	6.6	18.9	45.8	501.8	Medium	High
**Segovia**	2.4	4.7	15.8	39.9	509.4	Medium	Medium
**Badajoz**	2.3	4.6	11.7	46.8	569.7	Low	Low
**Ciudad Real**	2.3	3.6	18.8	46.7	1068.2	Low	Low
**Palencia**	2.3	4.5	22.2	43.4	535.1	Medium	Medium
**Zaragoza**	2.3	11.0	25.3	36.5	411.6	Medium	Medium
**Burgos**	2.2	5.9	23.4	48.2	558.6	Medium	Medium
**Coruña (A)**	2.2	7.5	8.9	37.8	840.5	Medium	Low
**Tarragona**	2.2	10.3	16.0	45.9	644.3	Medium	Medium
**Valladolid**	2.2	7.9	19.6	51.3	467.7	High	Medium
**Albacete**	2.1	6.6	21.3	43.1	356.1	Low	Low
**Huelva**	2.1	7.8	9.3	36.6	435.3	Low	Low
**Cádiz**	2.0	12.8	8.1	45.8	554.4	Medium	Low
**Cuenca**	2.0	5.6	21.4	32.0	385.6	Low	Low
**Girona**	2.0	5.8	16.2	49.0	541.2	High	High
**Granada**	2.0	10.8	10.1	38.2	1485.9	Medium	Low
**Murcia (Región de)**	2.0	12.0	15.1	53.4	609.0	Low	Low
**Navarra**	2.0	6.6	13.9	58.3	904.7	High	High
**Zamora**	2.0	6.0	18.0	35.5	425.6	Low	Low
**Alava**	1.9	11.8	18.3	55.3	696.1	High	High
**Asturias (Principado de)**	1.9	6.0	11.6	40.3	447.2	Medium	Medium
**Córdoba**	1.9	3.1	17.0	44.4	583.2	Low	Low
**Jaén**	1.9	5.7	16.7	50.5	384.2	Low	Low
**Sevilla**	1.9	11.3	13.4	49.7	606.7	Medium	Low
**León**	1.8	8.0	14.9	36.4	520.6	Medium	Medium
**Toledo**	1.8	8.2	17.3	36.4	354.2	Low	Low
**Alicante/Alacant**	1.7	16.6	11.2	57.8	253.6	Low	Low
**Guipúzcoa**	1.7	12.2	11.8	52.1	489.7	High	High
**Balears (Illes)**	1.6	3.8	6.4	45.6	644.1	Medium	High
**Guadalajara**	1.6	3.7	19.5	45.3	547.7	Medium	Low
**Pontevedra**	1.6	6.7	9.4	51.5	1703.1	Medium	Low
**Málaga**	1.5	16.7	8.6	41.4	270.0	Low	Low
**Valencia**	1.5	14.8	17.3	36.7	476.1	Medium	Medium
**Vizcaya**	1.4	12.9	10.0	49.4	1080.3	High	High
**Barcelona**	1.1	15.8	12.1	40.8	560.9	High	High
**Cantabria**	1.1	7.5	21.1	36.8	434.7	High	Medium
**Madrid (Comunidad de)**	1.1	26.7	10.4	41.4	638.1	High	High
**Palmas (Las)**	1.1	7.4	6.0	38.2	201.1	High	Medium
**Santa Cruz de Tenerife**	0.7	6.4	6.1	39.9	1077.6	Medium	Medium

Notes: * Deaths per 100 million vehicle-kilometres travelled, average 2002–2004

The mean proportion of high capacity roads was 10.9% (SD = 6.5); the mean proportion of heavy vehicles in total traffic was 13.5% (SD = 4.8); the mean total precipitation was 588.8 mm (SD = 294.4); and the mean proportion of consumers of alcohol was 43.9% (SD = 6.2). In all the provinces where RTI per 100 mvk exceeded 2.5 (a total of 10 provinces), the range of the proportion of high capacity roads (from 1.2 to 9.7%) was below the average for the 50 provinces, and the proportion of heavy vehicles with respect to total traffic, in the same 10 provinces (from 13.6 to 28.2%) was above the average for the 50 provinces. In 9 of the 10 provinces where the adjusted RTI death rate was lowest, the educational level was medium/high, although this pattern was not found for the variable 'variable income'.

According to simple linear regression analysis, the rate of RTI deaths per 100 mvk presented a statistically significant association in the following variables (p < 0.01): in a positive sense, for the proportion of heavy vehicles on the roads – i.e. for every one percentage point increase in the rate of heavy vehicles, there was an increase of 0.055% (95% CI 0.030 – 0.079) in that of road traffic deaths per 100 mvk; and inversely for the index of cultural and educational levels – i.e. the difference between the value of the coefficient for low educational level and that of the coefficient for high educational level was 0.68 (95% CI 0.31 – 1.04) (Table 2).

**Table 2:** Analysis of simple linear regression between various characteristics of the provinces and the road traffic injury mortality rate, adjusted per 100 million vehicle-kilometres travelled

**Variables**	**β**	**p**	**CI 95% β**	**R 2**
**% High capacity roads**	-0.055	>0.001	-0.082/-0.028	0.263
**% Heavy vehicles**	0.055	>0.001	0.030/0.079	0.295
**% Consum ers of alcohol**	0.011	0.339	-0.012/0.034	0.019
**Mean precipitacion (mm)**	-0.00018	0.983	-0.00068/0.00033	0.01
**Educational and culture level**				
Low	0.679	0.001	0.314/1.045	0.231
Medium	0.491	0.009	0.131/0.851	
High	0			
**Income**				
Low	0.284	0.156	-0.112/0.680	0.053
Medium	0.306	0.140	-0.104/0.716	
High	0			

After fitting the data by the multiple regression model, we examined a broader spectrum of variables that presented a statistically significant association with RTI mortality (Table 3). All other variables being equal, a higher proportion of kilometres available on high capacity roads, and a higher cultural and education level were associated with lower death rates due to RTI, while the opposite was true for the rate of alcohol consumers and the road traffic volume of heavy vehicles. The variables included in the model accounted for 55.4% of the variability in RTI mortality, of which 46% of the variability is related to contextual variables.

**Table 3:** Analysis of multiple linear regression between various characteristics of the provinces and the road traffic injury mortality rate, adjusted per 100 million vehicle-kilometres travelled

**Variables**	**β**	**p**	**CI 95% β**	**Cumulative R2**
**% High capacity roads**	-0.031	0.015	-0.056/-0.006	0.263
**% Heavy vehicles**	0.038	0.001	0.017/0.060	0.414
**% Consumers of alcohol**	0.02	0.019	0.004/0.037	0.455
**Educational and culture level**				
Low	0.484	0.003	0.172/0.797	0.554
Medium	0.336	0.035	0.025/0.684	
High	0			

## Discussion

Adjusting RTI deaths by the number of vehicle-kilometres travelled reveals the high variability of this cause of death in Spain, and its ecological relation with variables such as alcohol consumption, type of traffic (transit of heavy vehicles), social indicators (index of cultural and educational level) and the role of high capacity roads.

In any study, the possibility of ecological fallacy must be borne in mind; the observation of a relation, at the population level, between two variables does not necessarily mean that the same relation holds good at an individual level, although the potential remains that such a relation may describe differences between populations [26], and it does enable contextual variables to be analysed. The strong point of ecological studies in the analysis of variables related to RTIs, which are largely contextual, is that if their goal is to estimate the ecological effect of contextual variables, the most appropriate level of analysis for causal inference is an ecological one. This type of approach makes it possible to establish the foundations for determining and evaluating decision making and action taking in the field of public health, such as enforcing speed limits or improving infrastructure.

Although we were unable to analyse certain individual variables that are of enormous importance in RTI mortality, such as driver age, gender, speed, somnolence, the use made of safety measures, or the possible influence of the emergency services, alcohol consumption, at an ecological level, was included. Other ecological variables that are not controlled and that may influence or mediate the results include traffic density, speed limits, the design and layout of roads, and regional differences in systems used to record death rates. Seasonality might be thought to bias the results, due to the fact that significant increases in traffic occur during the summer; nevertheless, the method of adjusting per kilometre travelled rules out this effect. Future studies could examine the evolution of the degree of hazard associated with seasonal variations. Nevertheless, by means of adjusting for the number of vehicle-kilometres travelled, information was obtained on the significant role of the type of roads involved in RTI (this factor accounted for 26% of the variability measured) and the transit volume of heavy vehicles (which accounted for 15%), such that a high proportion of the RTI variability is explained.

The existence of geographic variability in RTI deaths is a phenomenon that has been described by Redondo-Calderon et al. [19] for the situation in Spain, and for that in the Netherlands by Van Beeck et al. [27]. These studies adjusted the data for the death rate, and reported variability statistics in line with those presented in our paper.

Frontal collisions frequently occur on secondary roads with a single lane in each direction, when one driver overtakes a slower vehicle and meets oncoming traffic; on the contrary, such events are unlikely to occur on high capacity roads where dividing barriers are installed [28]. In a DGT report published in 2003, information was given on the type of RTI in which deaths occur; 19% of the latter resulted from frontal collisions [20]. This finding is coherent with the results of the present study, in which we conclude that the lower the proportion of high capacity roads, the higher the adjusted rate of mortality. According to a DGT report, 81% of RTI deaths in 2004 occurred on roads [29]. The same report noted that in 18% of fatal injuries, at least one heavy vehicle was involved.

The differences in socioeconomic position and the association between the latter and RTIs have been highlighted in several studies, which take into account factors such as the type of vehicle used, availability of safety equipment and varying levels of exposure to risk environments [27,30]. In a follow up study carried out on an area of high RTI deaths in southern Spain, an association was found between higher educational level and the greater use made of safety belts, helmets and child-safety seats [31]; it should be borne in mind that a single factor, the use of the safety belt, reduces the risk of RTI death by 42% [32]; this casts further light upon the inverse relation between education level and RTI mortality found in our study at an ecologic level.

In the field of public health, an active role must be played in protecting the population against RTIs, by encouraging the elimination of the obsolete causal model by which RTIs are attributed exclusively to human conduct. Instead, we should foster the implementation of comprehensive measures to prevent accidents and regulate traffic. Such measures are costly, but effective in the medium and long term [18]. Although this was not the specific goal of our study, it can be concluded, in agreement with Hummel, that the promotion of policies of intelligent growth, by which compact, higher-density development, with the promotion of policies of intelligent growth, by which compact, higher-density development, with mixed land use, is implemented, would make it possible for spaces where people live, work, go to school, do their shopping, take part in leisure activities, etc., to be near each other. Such a situation would facilitate the options of walking, using a bicycle or taking public transport, and would encourage a closer approach to the principles of sustainable development [33].

Adjusting RTI deaths by the vehicle-kilometres travelled identifies the variability in this parameter and its relation with environmental risk factors. This denominator should be used in future studies whenever the sources of information are accessible; furthermore, reconsideration should be made of the conclusions drawn in studies using any other type of denominator. In addition, it is important to consider the risk factors arising from the environment, such as the socioeconomic level of the population, the capacity of existing road infrastructure and the type of traffic making use of it.

## Conclusion

Adjusting RTI mortality rates by the number of vehicle-kilometres travelled enables us to identify the high variability of this cause of death, and its relation with risk factors other than those inherent to human behaviour, such as the type of roads and the type of vehicles using them. Investment in improving the highway network and encouraging an increase in demand for public transport, on the basis of an increase in its geographic flexibility, economic competitiveness and capacity, are valid responses to demands for a reduction in RTI mortality rates, in addition to road safety campaigns and driver training schemes.

## Competing interests

The author(s) declare that they have no competing interests.

## Authors' contributions

All authors contributed to the design of the study. Revision of the different versions of the study protocol: FRR, EPM, AJP. Substantial contributions to the conception and design of the digital data record: FRR, EPM, AJP. Acquisition of data and quality control: FRR, EPM, AJP. Analysis and interpretation of data: FRR, EPM, AJP. All authors have read and approved the final manuscript.

## Pre-publication history

The pre-publication history for this paper can be accessed here:


